# Role of Melatonin in the Management of Substance Addiction: A Systematic Review

**DOI:** 10.7759/cureus.26764

**Published:** 2022-07-11

**Authors:** Arani Das, Manoj Prithviraj, Palani Selvam Mohanraj

**Affiliations:** 1 Physiology, All India Institute of Medical Sciences, Gorakhpur, IND; 2 Psychiatry, All India Institute of Medical Sciences, Gorakhpur, IND; 3 Biochemistry, All India Institute of Medical Sciences, Gorakhpur, IND

**Keywords:** benzodiazepine, sleep disturbance, addiction, substance abuse, melatonin

## Abstract

Recent evidence links melatonin hormone and its receptor to the etiology and behavioral manifestation of addiction. The role of exogenous melatonin in addiction treatment is still inconsistent and unclear. The present study aimed to review the literature on randomized clinical trials that evaluated the role of melatonin supplementation, compared to placebo, in the treatment of various substance addictions. The literature searches of relevant articles published in the English language in MEDLINE and Google Scholar databases were performed from inception up to May 2021. We included only randomized clinical trials investigating the effect of melatonin treatment, compared to placebo, on substance addiction-related parameters. Non-randomized clinical trials, observation studies, and animal studies were excluded. The risk of bias-2 was used to assess the quality of the studies. Of 537 articles, 12 randomized control trials (RCT) met our inclusion criteria. Studies have been conducted on substances of addiction including benzodiazepine (BZD), alcohol, nicotine, and opioids. Our results indicated that melatonin treatment had mixed results in improving sleep quality and was not found beneficial in BDZ cessation/discontinuation rate among patients with BDZ dependence. Sleep quality and mental health had improved by melatonin supplements in opioid addiction. In nicotine addiction, melatonin treatment showed effectiveness only on mood changes but not in performance tests. In patients with alcohol use disorder (AUD), melatonin treatment did not show any improvement in sleep quality. We found that the use of exogenous melatonin in substance addiction has mixed results which do not provide sufficient evidence, relative to randomized clinical trials, to establish its role.

## Introduction and background

Substance addiction is characterized by compulsive drug-seeking behavior, development of tolerance over prolonged use, and withdrawal symptoms in its absence [1]. Drug abuse has emerged as a serious concern, adversely affecting the physical and mental health of an individual and their socioeconomic well-being. According to World Health Organization (WHO) report, more than 35 million people are affected by substance addiction across the globe [2]. In India, about six million people require medical treatment for substance addiction every year [3]. Psychostimulant substances can affect several neural circuits which are involved in various cognitive functions such as reward, motivation, learning, memory, and decision making [4]. It can also disrupt the sleep-wake cycle and circadian rhythm, which are well documented in almost all types of substance addiction [5-9]. A study by Schierenbeck et al. showed that both stimulants (cocaine, amphetamine) and depressants (benzodiazepines, alcohol, and opiates) can lead to disruptions of sleep architecture [7].

Research on human studies revealed that core genes that regulate circadian rhythm are also important regulators of reward-related behaviors in substance abuse [10]. The associations between these circadian genetic alterations and addiction have also been reported in experimental animal studies, e.g., Garmabi et al. [11]. This suggests there might be a complex relationship between substance addiction and circadian rhythm abnormalities. However, the treatment options for sleep and circadian rhythm abnormalities associated with drug addiction are very limited. Conventional drugs such as benzodiazepines used in addiction treatment are prone to frequent abuse and are associated with withdrawal symptoms if discontinued. There is an increased interest to find a new pharmaceutical approach with less side effects. One of the candidate drugs that has shown some promising results in this respect is melatonin.

Melatonin is a neurohormone secreted from the pineal gland and widely recognized as a predominant synchronizer of circadian rhythms, reproduction, neurobehaviour, antioxidant status, and general immunity [12]. Melatonin exerts its biological effects via melatonin receptors (MT) 1 and 2. MT1 receptor subtype is identified in different brain regions, such as the prefrontal cortex, hippocampus, nucleus accumbens, and amygdala which are also associated with addiction-related behaviors [13].

Uz et al. have documented the presence of MT1 receptor expression in the dopaminergic system of the human and rodent brains [14]. In another animal study, Imbesi et al. showed prolonged treatment with antidepressants and cocaine was associated with brain region-specific alteration in the quantity of melatonin receptor mRNA levels [13]. All these findings indicate melatonin might have a role in drug addiction. Few studies have shown that exogenous melatonin can be useful in the management of withdrawal symptoms and relapse of certain drugs of abuse [15-18]. Vengeliene et al. have reported that the administration of melatonin modulates alcohol-seeking and relapse behaviors in rats [19]. Takahashi et al. showed exogenous melatonin reduces the relapse-like behavior in cocaine-addicted rats [20]. However, its role in addiction and related sleep and/or circadian rhythm disorders is still unclear. Some of the randomized placebo control trials concluded that melatonin intervention showed no improvement in addiction-related sleep abnormality [21] or withdrawal symptoms of benzodiazepine dependence [22]. To the best of our knowledge, there is no comprehensive systematic review on the melatonin intervention for the improvement of withdrawal symptoms and drug dependence in different types of addiction. Thus, with accumulating evidence, we performed a systematic review to investigate the role of exogenous melatonin in addiction and related symptoms.

## Review

Methods

This systematic review was conducted according to Preferred Reporting Items for Systematic Reviews and Meta-Analyses (PRISMA) guidelines [23]. The objective was to explore the effectiveness of melatonin therapy/supplementation in the treatment of substance addiction with respect to treatment-related outcomes. The patient/population, intervention, comparison, and outcomes (PICO) model for the study is as follows: population - patients diagnosed with any form of substance addiction; intervention - melatonin/melatonin agonist as therapy/supplementation; comparison - any pharmacological/non-pharmacological management; outcome - complete or partial recovery from any form of addiction/substance abuse-related disorders/withdrawal symptoms. The review protocol was registered and can be accessed with registration id - CRD42021252745 in the international prospective register of systematic reviews (PROSPERO).

Search Strategy

A systematic search of the literature was conducted in Medline/PubMed and Google Scholar from inception to May 2021. We included only those studies in the English language. We used the following search strategy. PubMed: ("melatonin"{MeSH terms} OR "melatonin"{All Fields} OR "melatonin s"{All Fields} OR "melatonine"{All Fields} OR "melatonins"{All Fields}) AND ("substance-related disorders"{MeSH terms} OR ("substance related"{All Fields} AND "disorders"{All Fields}) OR "substance-related disorders"{All Fields} OR ("substance"{All Fields} AND "related"{All Fields} AND "disorders"{All Fields}) OR "substance-related disorders"{All Fields} OR ("drug"{All Fields} AND "abuse"{All Fields}) OR "drug abuse"{All Fields} OR (("drug"{All Fields} AND "dependence"{All Fields}) OR "drug dependence"{All Fields}) OR ("substance withdrawal syndrome"{MeSH terms} OR ("substance"{All Fields} AND "withdrawal"{All Fields} AND "syndrome"{All Fields}) OR "substance withdrawal syndrome"{All Fields} OR ("drug"{All Fields} AND "withdrawal"{All Fields}) OR "drug withdrawal"{All Fields})). Google Scholar: allintitle: melatonin AND (addiction OR drug OR abuse OR substance).

Selection Procedure

The articles primarily examined exogenous melatonin as the main therapy or as a supplement to improve the treatment outcomes such as withdrawal symptoms or relapse rate in drug addiction. Studies that have examined the role of melatonin in the management of BZD discontinuation after long-term therapeutic use have also been included. Long-term therapeutic use means those patients who have been taking BZD as a therapeutic agent over a period of time. Treatment could involve the administration of exogenous melatonin or melatonin agonists. We included randomized controlled trials (RCTs) only. The screening and selection process was conducted using the open-access online tool CADIMA version 2.2.3 (Quedlinburg, Germany: Julius Kühn Institute) [24]. The study screening was done by two independent authors and wherever there was a conflict of opinion; it was screened by a third reviewer to maintain the objectivity of the screening procedure. The selection was first based on title and abstract and then the full text was screened subsequently. Studies with irrelevant populations, using endogenous melatonin levels, or without any control group/placebo as a comparator were excluded. The data were collected independently by two authors from the included studies.

Quality Assessment

**Figure 1:**
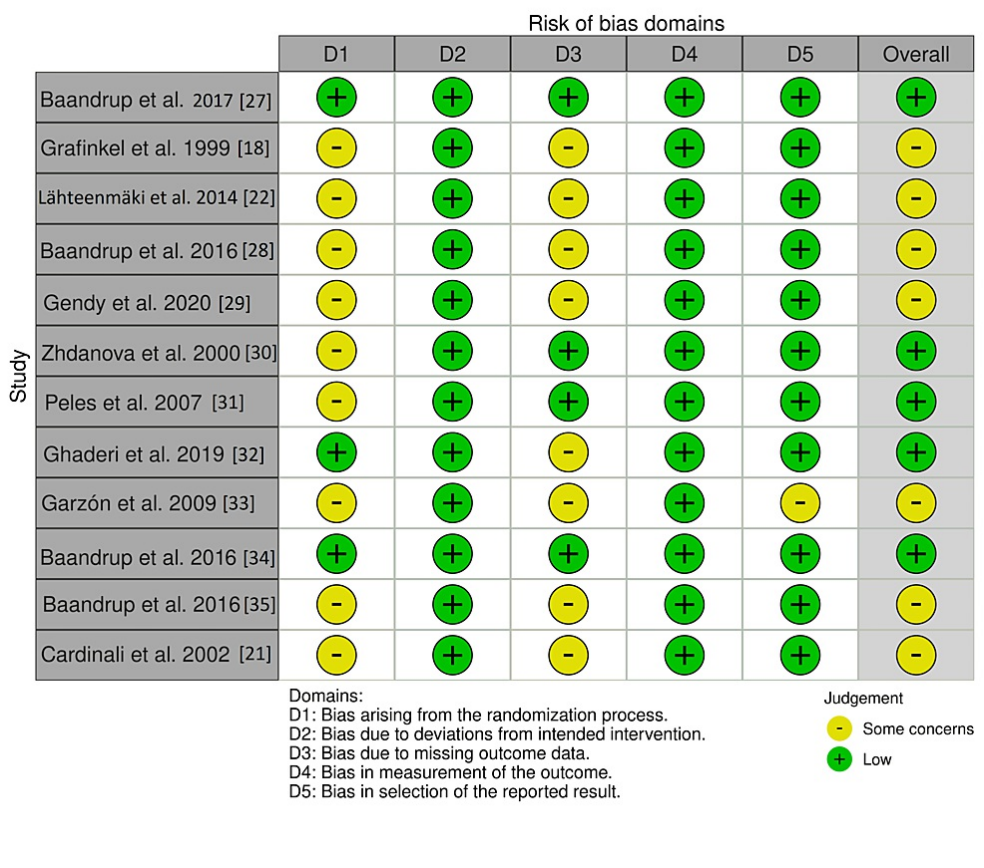
Risk of Bias summary for included studies

**Figure 2:**
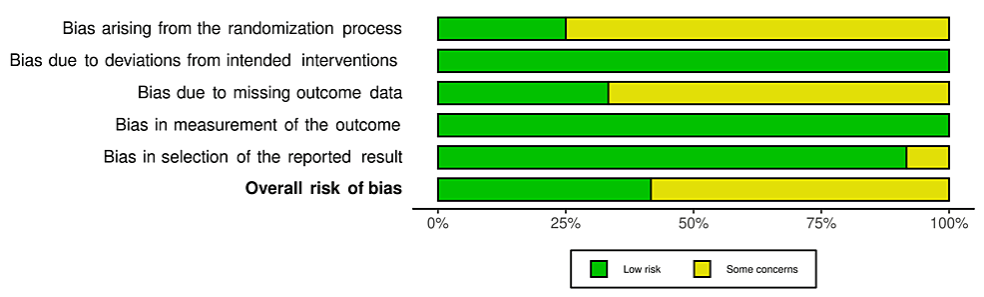
Risk of Bias graph for included studies

The quality of the included studies was assessed using the Cochrane tool to assess the risk of bias for randomized controlled trials [25]. The studies were classified as high risk of bias, low risk of bias, and unclear risk of bias. The risk of bias summary and graph was generated using the online tool Risk-of-bias VISualization (robvis; Luke McGuinness, Bristol Medical School, Bristol, Uk) [26] and the summary of studies is shown in Figures 1, 2 [18,21,22,27-35].

Results

**Figure 3:**
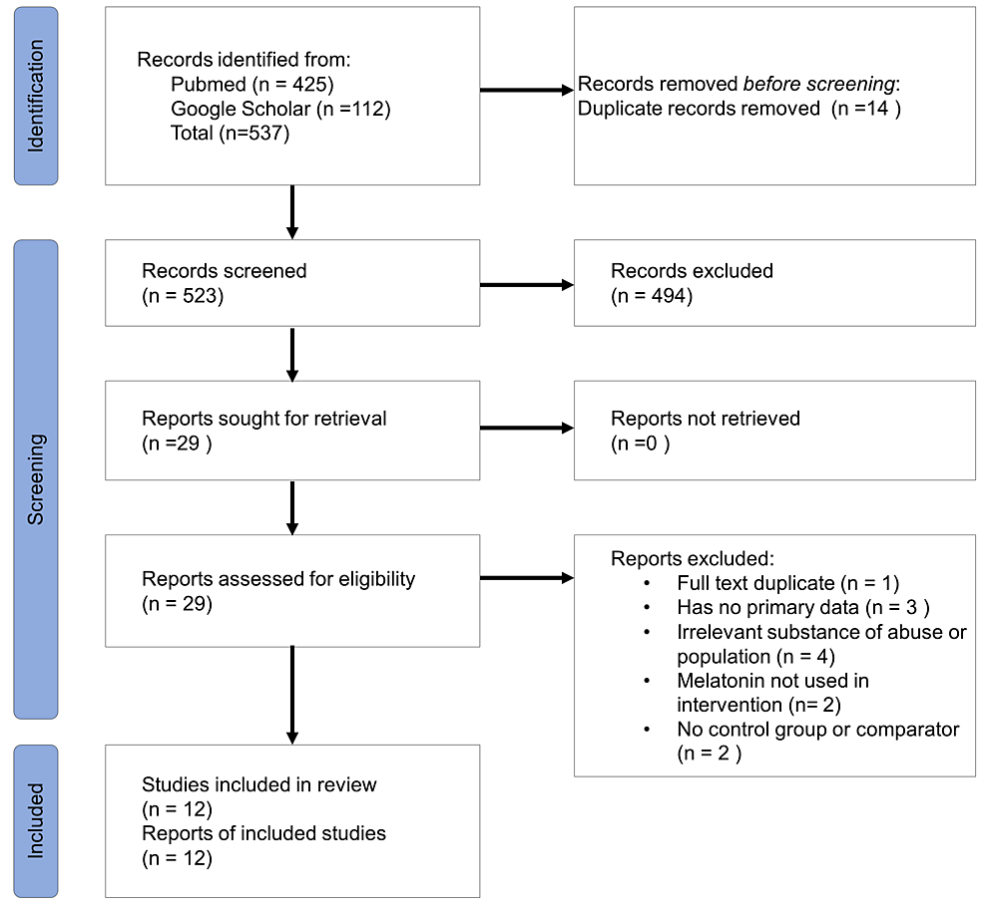
PRISMA flow diagram PRISMA: Preferred Reporting Items for Systematic Reviews and Meta-Analyses

The search strategy identified 537 articles including 425 in the PubMed database and 112 from Google Scholar. After duplicate removal and merging of results from both databases, a total of 523 articles were identified. PRISMA flow chart of the search process is shown in Figure 3.

After screening the title and abstract, 29 articles were eligible for full-text screening. Among them, 17 were excluded due to the following reasons: irrelevant substance of abuse or population; melatonin not used in Intervention; no control group or comparator used. Finally, a total of 12 articles were included in this systematic review. 

Study Characteristics

The included studies were conducted in eight different countries such as Denmark [27,28,34,35], Israel [18,31], Iran [32], USA [30], Canada [29], Argentina [21], Spain [33], and Finland [22]. Studies have been conducted on various substances of addiction including benzodiazepine [18,21,22,27,28,31,33-35], alcohol [29], nicotine [36] and opioid [32]. The sample size of the included studies ranges from 12 to 92 subjects (Table 1).

**Table 1:** Basic characteristics of included studies *Data are presented as mean ± SD or median (range). BZD: benzodiazepine; RCT: randomized control trial; I: intervention group; C: placebo group, MMT: methadone maintenance treatment

Reference	Country	Population	Substance abuse	Sample size total (I/C)	Age, years*	Gender	Design
Baandrup et al. [27]	Denmark	Schizophrenia or bipolar disorder	BZD	80 (40/40)	I: 47.4 ± 8.6 C: 49 ± 12.1	I: M21, F 19 C: M 24, F16	RCT
Grafinkel et al. [18]	Israel	Insomnia	BZD	34 (18/16)	I: 69 ±11 C: 68 ±16	I: M14, F4 C: M 11, F5	RCT
Lähteenmäki et al. [22]	Finland	Insomnia with long-term BZD use	BZD	92 (46/46)	I: 66.5 (61- 72) C: 65 (60-70)	I: M19, F 27 C:M 12, F34	RCT
Baandrup et al. [28]	Denmark	Schizophrenia or bipolar disorder	BZD	55 (28/27)	I: 48.8 ± 7.1 C: 49.1 ± 12.2	I: M14, F14 C: M15, F12	RCT
Gendy et al. [29]	Canada	Alcohol use disorder with Insomnia	Alcohol	60 (30/30)	Not mentioned	I: M23, F7 C: M23, F7	RCT
Zhdanova and Piotrovskaya [30]	USA	Smoker (20 cigarettes per day)	Nicotine	12	27.9 ± 3.8	M6, F6	RCT, crossover
Peles et al. [31]	Israel	BZD withdrawal in MMT	BZD	80 (40/40)	42.6 ± 1.2	M56, F24	RCT, crossover
Ghaderi et al. [32]	Iran	MMT	Opioid	54 (26/28)	25-70	M54	RCT
Garzón et al. [33]	Spain	Insomnia	BZD	22	>65	M7, F15	RCT, crossover
Baandrup et al. [34]	Denmark	Schizophrenia or bipolar disorder with long-term BZD use	BZD	86 (42/44)	21-74	I: M23, F19 C: M25, F19	RCT
Baandrup et al. [35]	Denmark	Schizophrenia or bipolar disorder with long-term BZD use	BZD	48 (20/28)	I: 47.7 ± 8.2 C: 45.9 ± 10.3)	I: M 11, F 9 C: M18, F10	RCT
Cardinali et al. [21]	Argentina	Insomnia with long-term BZD use	BZD	45 (24/21)	I: 70.1 ± 16.8 C: 71.0 ± 7.3	I: M 5, F 19 C: M4, F17	RCT

Different formulations of melatonin such as immediate-release, controlled-release, and prolonged-release had been used. The dose range of melatonin in the included studies varies widely between 0.3 mg and 10 mg per day (Table 2).

**Table 2:** Details of melatonin intervention PRM: prolonged-release melatonin; CRM: controlled-release melatonin; IRM: immediate-release melatonin; I: intervention group; C: placebo group

Reference	Melatonin dosage	Administration	Treatment Duration	Follow-up duration
Baandrup et al. [27]	2-mg CRM	Once daily	12 weeks	6 months
Grafinkel et al. [18]	2-mg CRM	Once daily	12 weeks	6 months
Lähteenmäki et al. [22]	2-mg CRM	Once daily	4 weeks	5 months
Baandrup et al. [28]	2 mg PRM	Once daily	24 weeks	No follow-up
Gendy et al. [29]	5 mg	Once daily	4 weeks	No follow-up
Zhdanova et al. [30]	0.3 mg	Single dose	2 day (I: 1 day, C: 1 day)	No follow-up
Peles et al. [31]	5 mg	Once daily	12 weeks (I: 6 weeks, C: 6 weeks)	No follow-up
Ghaderi et al. [32]	10 mg	Once daily	12 weeks	No follow-up
Garzón et al. [33]	5 mg	Once daily	4 months (I: 2months, C: 2months)	No follow-up
Baandrup et al. [34]	2 mg	Once daily	24 weeks	No follow-up
Baandrup et al. [35]	2-mg PRM	Once daily	24 weeks	No follow-up
Cardinali et al. [21]	3 mg IRM	Once daily	6 weeks	No follow-up

The main outcome measure of the included study was the assessment of sleep quality. The other common outcome measures were depression, anxiety, BZD discontinuation rate, and withdrawal symptoms. Out of 12 studies, nine studies have evaluated the efficacy of melatonin in BZD addiction. Among these, three studies have shown a beneficial effect on outcome measures, five studies did not show any beneficial effect, and one study has reported improvement only in sleep quality but not on sleep efficiency. In a study on nicotine addiction melatonin is found to be beneficial in improving the subjective test score such as visual analog scale (VAS) but no improvement was shown in the performance tests. Melatonin did not show any beneficial effect in a study conducted on alcohol addiction. Another study on opioid addiction showed that melatonin has beneficial effects on the outcome measures (Table 3).

**Table 3:** Study outcome and main findings BACS: brief assessment of cognition in schizophrenia; BWSQ: benzodiazepine withdrawal symptom questionnaire; PSQI: Pittsburgh Sleep Quality Index; BDI: Beck Depression Inventory; BAI: Beck Anxiety Inventory; VAS: visual analog scale; FCRT: four-choice reaction time; SART: simple auditory reaction time; CES-D: Center for Epidemiologic Studies Depression Scale; NHSMI: Northside Hospital Sleep Medicine Institute test; GDS: Geriatric Depression Scale; GAS: Goldberg Anxiety Scale

Reference	Outcome	Main findings
Baandrup et al. [27]	Global cognitive performance (BACS), quality of life (WHO-five well-being index), subjective well-being (neuroleptic treatment scale), and psychological functioning (personal and social performance scale)	Melatonin did not show any additional effect on cognition, quality of life, subjective well-being, and psychosocial functioning during BDZ dose reduction
Grafinkel et al. [18]	Benzodiazepine discontinuation rate and Subjective sleep-quality score	Melatonin facilitated discontinuation of BDZ usage and improved subjective sleep quality compared to placebo
Lähteenmäki et al. [22]	Benzodiazepine abstinence rate, Reduction of BZD usage, and BZD withdrawal symptoms (BWSQ)	Melatonin did not show any superior effect over the placebo
Baandrup et al. [28]	Sleep efficiency (polysomnography) and sleep quality (PSQI global score)	Melatonin did not affect sleep efficiency but improved sleep quality
Gendy et al. [29]	Sleep quality (PSQI), depression (BDI), anxiety (BAI)	Melatonin did not show any improvement in sleep quality, depression, or anxiety scores over the placebo group
Zhdanova et al. [30]	Self-reported ratings of mood, sleepiness, and cigarette craving using 17 VAS and performance tests (FCRT and SART)	Melatonin improved VAS ratings of mood, sleepiness, and cigarette craving but did not show improvement on the performance test.
Peles et al. [31]	Sleep quality (PSQI), depression (CES-D)	Melatonin did not show any improvement in sleep quality and depression score
Ghaderi et al. [32]	Sleep quality (PSQI), depression (BDI), anxiety (BAI), erectile functions, and metabolic profile	Melatonin supplement showed beneficial effects on sleep quality, depression, anxiety, erectile functions, and metabolic profile
Garzón et al. [33]	Sleep quality (NHSMI), depression (GDS), and anxiety (GAS)	Melatonin improved sleep quality, depression, and anxiety scores
Baandrup et al. [34]	Benzodiazepine dosage, cessation rate, and benzodiazepine withdrawal symptoms (BWSQ-2)	Melatonin did not affect the reduction of benzodiazepine dosage, cessation rate, and benzodiazepine withdrawal symptoms
Baandrup et al. [35]	Circadian rest-activity cycles (actigraphy measurement)	Melatonin stabilizes circadian rest-activity cycles during benzodiazepine discontinuation
Cardinali et al. [21]	Sleep quality (quality of morning freshness, daily alertness, sleep quality and readiness to fall asleep, daily sleep onset and offset time)	Melatonin did not show any effect on sleep quality

Discussion

To the best of our knowledge, the current review is the first to investigate the role of exogenous melatonin in substance addiction. The studies included in this review show inconclusive evidence as there is less number of studies on each type of substance addiction. Melatonin treatment showed mixed results in improving sleep quality but did not show any beneficial role on BZD cessation/discontinuation rate among patients on long-term benzodiazepine use. Sleep quality and mental health have improved by melatonin supplement in opioid addiction. In nicotine addiction, melatonin treatment showed improvement only in mood changes but not in performance tests. In patients with alcohol use disorder (AUD), melatonin treatment did not show any improvement in sleep quality.

Benzodiazepine Abuse

BZDs are one of the most commonly used drugs in the treatment of insomnia. It is also used in a wide range of disorders such as anxiety, depression, and other substance use disorders and for behavioral symptoms such as irritability, agitation, violent behavior, and emotional lability seen in psychosis spectrum disorder and mood disorders [36]. The use of benzodiazepines is usually suggested for the shortest duration of time as required but in many cases, patients tend to prolong the use of BZD beyond the prescribed duration due to its addictive potential [37]. Long-term BZD use is also associated with the risk of cognitive dysfunction, excessive sedation, and dementia [38]. Patients with BZD dependence find it difficult to discontinue the same due to fear of withdrawal symptoms, the most common being sleep disturbances. Long-term BZD use is also associated with a decrease in melatonin synthesis, and receptor binding and alters circadian sleep rhythm [39,40]. Hence melatonin might be an alternative to benzodiazepine due to its lack of addictive potential.

In this review, nine studies have been identified in which the efficacy of melatonin was studied in the management of BZD discontinuation. Among these studies, mixed results have been reported regarding the effects of melatonin. Four studies found improvement in sleep quality while two studies did not show any improvement. Two studies that evaluated the BZD cessation or discontinuation rate did not show any beneficial effect of melatonin. In another study, melatonin was not beneficial in improving cognition, quality of life, subjective well-being, and psychosocial functioning during BDZ dose reduction. Most of the studies which found melatonin as beneficial had sleep quality as a primary outcome measure which is in line with the physiological action of melatonin. In other studies, which evaluated benzodiazepine cessation or improvement in withdrawal symptoms, melatonin did not show any additional benefit. The probable factors could be differences in duration of BZD use, dosages, pharmacokinetics, differences in outcome measurement scales, and associated psychiatric comorbidity. Studies that used controlled-release or prolonged-release formulation had shown beneficial effects as compared to other studies that used immediate-release formulation which may be explained by its very short half-life. The control of BZD withdrawal symptoms involves a complex neurophysiological mechanism that may not fall under the scope of melatonin and hence limiting its use in the management of withdrawal symptoms beyond sleep quality. The current evidence is still inconclusive about the role of melatonin in terms of BZD discontinuation or management of its withdrawal symptoms. Chronic BZD use leads to cognitive dysfunction. A study that examined the effect of melatonin on cognitive functioning while facilitating BZD discontinuation concluded that it doesn't have any additional benefit [27]. This finding contrasts with the previous literature that melatonin enhances cognitive functioning due to its anti-inflammatory and neuroprotective properties [41]. It is still debatable whether cognitive dysfunction due to chronic BZD use is reversible. Hence more studies with higher doses of melatonin and longer duration are required to explore the potential use of melatonin as a cognitive enhancer in patients with long-term BZD use.

Alcoholism

Sleep disturbance is an extremely common and persistent problem among alcohol use disorder patients [42]. This may be multifactorial including depression, general disturbance in physiological sleep patterns, or circadian rhythm disturbances [43]. Chronic alcohol consumption has been found to alter melatonin production and function and can also lead to a delay in the peak rise of melatonin [44]. The disturbance in normal sleep patterns among AUD patients is a huge barrier to successful treatment as there is a tendency to relapse to alcohol use [45]. Pharmacological treatment with benzodiazepines for sleep disturbances in AUD patients is commonly associated with abuse potential. An alternative treatment for insomnia among AUD patients is the need of the hour. Even though there is no substantial evidence for its efficacy, melatonin is being prescribed as a treatment or supplement with other treatment options for treating sleep disorders in AUD patients.

In the literature, we found very few studies that evaluated the use of melatonin/melatonin agonists in alcohol use disorders. In a study included in this review, Gendy et al., the role of melatonin was assessed to treat sleep problems in patients with AUD [29]. It has been reported that there was a reduction in the global Pittsburgh Sleep Quality Index (PSQI) score in both melatonin and placebo groups but there was no significant difference between the groups. This finding can be explained by the fact that the treatment is of a very short duration of four weeks which may not be sufficient to reverse the damage done by chronic alcohol use. In contrast to this trial's results, some studies which are not included in this review showed positive results. Grosshans et al. have shown that agomelatine, a melatonin agonist, improved the sleep quality in alcohol-dependent patients associated with severe insomnia after six weeks [46]. In another open-label study, Ramelteon, a melatonin agonist, improved the sleep quality as assessed using Insomnia Severity Index and a sleep diary [47]. Even though there is a significant improvement in sleep quality in these studies there was no placebo/control group to compare and attribute the results. Till now only very few placebo-controlled randomized trials are available in the literature; it is difficult to conclude the effectiveness of melatonin in AUDs.

Nicotine Addiction

Nicotine withdrawal symptoms include anxiety, stress, irritability, and associated sleep disturbance [48]. Melatonin might improve the symptoms due to its effect on multiple physiological functions including sleep promotion [49]. Sleep disturbances are associated with the acute cessation of nicotine or as a side effect of long-term nicotine addiction [50]. Zhdanova and Piotrovskaya studied the usefulness of melatonin in attenuating the withdrawal symptoms of nicotine addiction [30]. A single oral dose of 0.3 mg of melatonin was administered to regular smokers after 3.5 hours of nicotine cessation. They found that melatonin reduced self-reported ratings of mood using a visual analog scale but does not affect the responses on the performance tests significantly. This study concluded that melatonin may help in reducing the mood changes associated with nicotine withdrawal. A single dose of melatonin with a very low concentration was used in this study which may be the reason for the inconclusive result of this trial. Future studies with higher dose and longer duration in large numbers of patients are required to evaluate the effect of melatonin on Nicotine addiction. Currently, there is not enough evidence in the literature to justify its use in the treatment of nicotine withdrawal.

Opioid Addiction

Opioid dependence is commonly associated with sleep disturbances which lead to a high prevalence of benzodiazepine abuse [51,52]. In this review, a study by Ghaderi et al., the effect of melatonin on sleep quality, depression, and anxiety among patients under methadone treatment for opioid dependence was assessed [32]. PQSI significantly decreased, whereas the Beck Depression Inventory index and Beck Anxiety Inventory index had significantly increased in the melatonin group compared to placebo. This study showed that taking melatonin supplement is beneficial in improving both sleep quality and mental health among patients under MMT. Another study by Peles et al. conducted a crossover randomized clinical trial to study the effectiveness of melatonin for sleep disturbances that are associated with benzodiazepine withdrawal among subjects who are in methadone maintenance treatment [31]. There was no difference in sleep quality among those who discontinued benzodiazepine in both interventional and placebo groups. This study concluded that melatonin had no significant effect on improving sleep quality or benzodiazepine discontinuation rate among patients under MMT. Both of the studies did not evaluate the effect of melatonin supplementation for a longer duration and did not correlate the effects with melatonin levels. Thus, the available evidence may not be sufficient to conclude a role of melatonin supplementation in the treatment of opioid dependence.

Other Drugs of Abuse

Even though there are several other drugs with abuse potential, we did not find any placebo-controlled RCTs in human volunteers in our literature search addressing role of melatonin in its management. Few animal studies have reported the role of exogenous melatonin in stimulant drugs like cocaine addiction. In an animal study, melatonin was shown to reduce relapse-like behavior [20] and in another study, it was shown to decrease cocaine-induced locomotor sensitization and cocaine-conditioned place preference [53]. Veschsanit et al. have reported promising role of melatonin treatment in reverting methamphetamine-induced learning and memory impairment and neuronal alteration [54]. Further studies are required to evaluate the role of exogenous melatonin in its management.

## Conclusions

The few RCTs that are available in the literature that evaluated the use of melatonin in substance addiction have shown mixed results which do not provide sufficient evidence to establish its role or its clinical recommendation. One of the reasons for mixed results is that the dose and duration of exogenous melatonin treatment varies in each study, which may lead to differences in the efficacy of treatment. Hence there is a necessity for more placebo-controlled double-blind RCTs with a larger number of subjects, a longer duration of treatments reviewed in this topic. These studies should also aspire to study the effect of melatonin on relapse rate to establish the role of melatonin in the management of substance use disorder and its withdrawal symptoms apart from its role in improving sleep-related parameters.
